# Voiding symptoms obtained by open versus directed anamnesis as predictors of voiding dysfunction in women

**DOI:** 10.1590/S1677-5538.IBJU.2018.0556

**Published:** 2019-09-02

**Authors:** Juan Pablo Valdevenito, José Flores, Rodrigo Guzman Rojas, Valentin Manriquez, Leandro Arribillaga, Juan de Benito

**Affiliations:** 1 Department of Urology, Urodynamics Unit, Hospital Clínico Universidad de Chile, Santiago, Chile;; 2 Department of Obstetrics and Gynecology, Female Pelvic Floor Unit, Hospital Clínico Universidad de Chile, Santiago, Chile;; 3 Centro Urológico Profesor Bengió, Córdoba, Argentina;; 4 Department of Urology, Instituto Modelo de Cardiología SRL, Córdoba, Argentina

**Keywords:** Urination, Women, Retrospective Studies

## Abstract

**Objectives:**

To determine the differences between voiding symptoms obtained by open anamnesis (VS-Open) versus voiding symptoms obtained by directed anamnesis (VS-Directed) to predict voiding dysfunction in women.

**Materials and Methods:**

Retrospective study of women with prior anti-incontinence surgery evaluated during 5 years. In a standardized clinical history taking, each patient was asked to answer question number five of the UDI-6 questionnaire (“Do you experience any difficulty emptying your bladder?”). If the answer was positive, the following voiding symptoms spontaneously described by the patient were documented: slow urine stream, straining to void, intermittent stream and feeling of incomplete bladder emptying, which were considered VS-Open. If the answer to this question was negative or if the patient had not reported the four voiding symptoms, she was asked in a directed manner about the presence of each o Ninety-one women are analyzed. Eighteen patients presented voiding dysfunction (19.8%), There was a statistical association between voiding dysfunction and the presence of any VS-Open (p = 0.037) and straining to void obtained by open anamnesis (p = 0.013). Sensitivity, specificity, PPV, NPV, positive likelihood ratio and negative likelihood ratio, respectively, were 44.4% and 27.8%, 80.8% and 94.5%, 36.3% and 55.6%, 85.5% and 84.1%, 2.324 and 5.129, and 0.686 and 0.764. There was no statistical association between voiding dysfunction and VS-Directed.

**Conclusions:**

VS-Open may predict better voiding dysfunction than VS-Directed in women.

## INTRODUCTION

Large epidemiologic studies have demonstrated that the prevalence of voiding symptoms in women ranges between 14.9 and 19.5%, and that these are generally related to storage symptoms (1, 2). The NICE (The National Institute for Health and Care Excellence, U.K.) guidelines for management of urinary incontinence in women confers value to the presence of “symptoms suggestive of voiding dysfunction” and recommends the performance of a multichannel urodynamics assessment before undergoing anti-incontinence surgery in women presenting with such symptoms (3).

Several studies have aimed at correlating voiding and/or post-micturition symptoms with voiding dysfunction in women, and have found difficulties at establishing such correlation. On one hand, there is a lack of consensus regarding the definition and diagnosis of voiding dysfunction in females (4), and there are studies that use only one criterion, such as either the urinary flow rate decrease or the increased post-void residual volume (PVR) (5-8). On the other hand, symptoms may be retrieved either through a medical interview (5, 6, 9-11) or through the implementation of standardized questionnaires (1, 2, 7, 12), thus generating variations in their value to predict voiding dysfunction.

In their most recent terminology report, the International Urogynecological Association (IUGA) and the International Continence Society (ICS) define voiding dysfunction as an abnormally slow and/or incomplete voiding and recommend it is studied with the use of uroflowmetry and PVR measurement, although there is still no consensus on the values that are considered as abnormal (13).

The purpose of the present exploratory work is to study whether the voiding symptoms are predictive for voiding dysfunction in women in accordance with the definition of the main international societies, and to define whether there are differences between voiding symptoms obtained by open anamnesis (VS-Open) versus voiding symptoms obtained by directed anamnesis (VS-Directed).

## MATERIALS AND METHODS

We retrospectively reviewed an electronic database of patient’s medical records in a university referral center. Patient information was collected and entered into a database at the time of history taking, and before conducting urodynamics according to ICS and IUGA definitions and recommendations (13-15). All patients provided informed consent for the use of their clinical information in research studies, and the confidentiality of the data was guaranteed. The project was approved by the Institutional Scientific Ethics Committee of our institution.

As part of a standardized clinical history procedure performed before every urodynamic study by the two urologist directly involved, each patient was asked to answer question number five of the Urogenital Distress Inventory Short Form Questionnaire (UDI-6) (“Do you experience any difficulty emptying your bladder?”) (16). If the answer was positive, the following voiding symptoms spontaneously described by the patient were documented: 1) slow urine stream, 2) straining to void, 3) intermittent stream (intermittency) and 4) a feeling of incomplete bladder emptying (according to the ICS, incomplete bladder emptying is a post-micturition symptom) (14). Any or all of the symptoms expressed spontaneously were considered VS-Open. If the answer to this question was negative or if the patient had not spontaneously reported experiencing the four voiding symptoms, she was asked in a directed manner about the presence of each of them. These symptoms were considered VS-Directed (VS-open were always described as VS-Directed subsequently). Symptoms were considered as either present or absent, with no severity stratification. All women with prior anti-incontinence surgery during 5 consecutive calendar years were selected, for being a group with a higher likelihood of presenting voiding dysfunction. The following exclusion criteria were applied: 1) pelvic organ prolapse over stage II, 2) “urethrolysis” surgery prior to the testing, 3) use of uroselective drugs, 4) neurological diseases, 5) bladder pain syndrome and 6) history of pelvic radiotherapy.

Urodynamic testing was performed in accordance with the recommendations of the ICS (15). First, a non-invasive uroflowmetry was performed in private and the PVR was measured through catheterization; the procedure was repeated in those patients presenting abnormal voiding testing or voiding volume <150 mL (until a proper volume was obtained). Subsequently, interactive filling cystometry was performed. A double lumen 6F urethro-vesical catheter was used for bladder filling and intravesical pressure measurement and a rectal 8F balloon catheter was used for abdominal pressure measurement. External pressure transducers were positioned at the upper edge of symphysis pubis and the system was zeroed to atmospheric pressure. Room temperature 0.9% saline solution was infused at a rate of 70 mL/min. Pressure transmission was assessed with coughing at the beginning and at the end of each testing, every 1 minute, during the complete testing and before and after each major event, in order to correct artifacts immediately; this was the only method used to provoke detrusor overactivity. The stress test was conducted in a standardized and stepped manner, with the use of progressively increasing cough intensity, following successive stages in case of not evidencing urodynamic stress incontinence: 1) with 300 mL infused in the sitting position, 2) with 300 mL infused in standing position and 3) at the maximum cystometric capacity in standing position (with the corresponding change of the position of the transducers). In patients with maximum cystometric capacity of less than 300 mL, it was generally conducted at capacity in the sitting and the standing positions. The pressure-flow study was performed in private. Finally, the PVR was measured through the urethro-vesical catheter.

The repeated presence of a maximum flow rate less than or equal to 12 mL/s and/or a PVR higher than 100 mL were considered as voiding dysfunction. The following was defined in the pressure-flow analysis: 1) bladder outlet obstruction was defined as a maximum flow rate ≤12 mL/s in association with detrusor pressure at maximum flow rate ≥25cm H_2_O (17); 2) reduced detrusor contractility was defined as a maximum flow rate ≤12 mL/s in association with a detrusor pressure at maximal flow rate ≤10cm H_2_O modified from Gotoh et al. (18) and 3) mixed voiding dysfunction was defined as a maximal flow rate ≤12 mL/s in association with a detrusor pressure at maximal flow rate between 11 and 24cm H_2_O), with a concordant free uroflowmetry in all cases.

**Figure 1:**
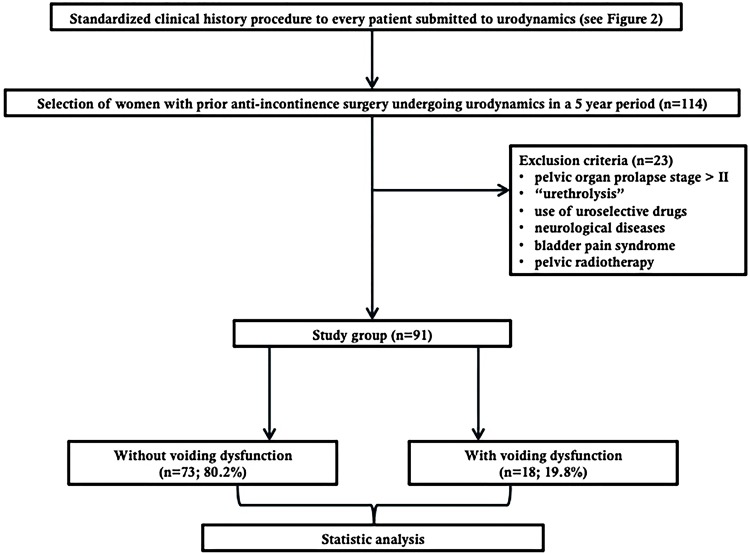
Flowchart of “Material and Methods”.

**Figure 2:**
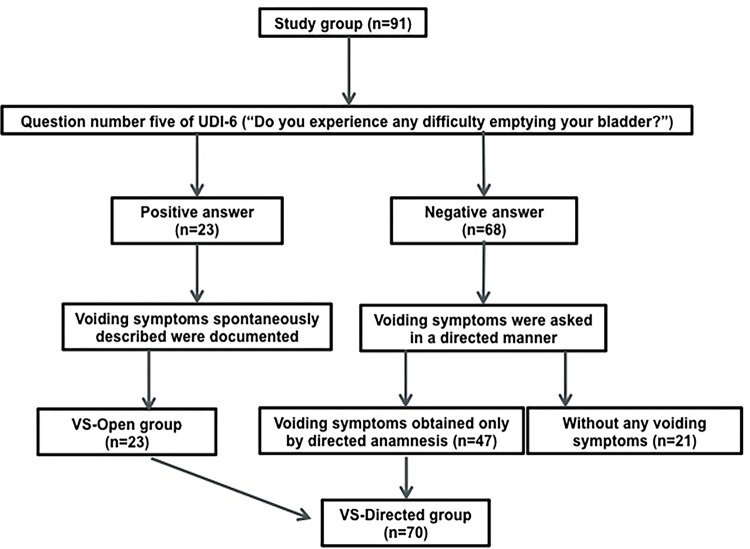
Flowchart of the standardized clinical history procedure performed before every urodynamic study (UDI-6: Urogenital Distress Inventory Short Form Questionnaire).

The Chi square test or the Fisher’s exact test were used to evaluate statistically significant association between voiding dysfunction and the presence of any of the VS-Open and VS-Directed. The procedure was applied likewise for each one of the symptoms individually. In case of obtaining a statistically significant result (p <0.05), the sensitivity, specificity, positive predictive value (PPV), negative predictive value (NPV), accuracy and positive and negative likelihood ratio and strength of agreement were calculated using Cohen’s kappa coefficient. Data were processed with Stata 12.1 program® (StataCorp, 2012). Figure-1 shows a flowchart of the “Material and Methods” and Figure-2 shows a flowchart of the standardized clinical history procedure performed before every urodynamic study.

## RESULTS

From a total of 114 women with prior anti-incontinence surgery undergoing urodynamics, 23 were excluded (6 for having pelvic organ prolapse over stage II, 5 for prior “urethrolysis”, 6 for using uroselective drugs, 5 for neurological diseases and 1 due to bladder pain syndrome), and 91 patients underwent the analysis. All patients were evaluated 6 or more months after the anti-incontinence surgery. Table-1 displays the clinical history of the patients. Twenty-three patients had VS-Open, 70 patients had VS-Directed (including the 23 patients with VS-Open) and only 21 patients didn´t have any kind of voiding symptoms (23.1%). Table-2 shows urodynamic diagnoses of the patients according to urinary incontinence symptoms. Of the total, 18 patients presented voiding dysfunction (19.8%): 13 had bladder outlet obstruction, 3 had reduced detrusor contractility and 2 had mixed voiding dysfunction. There were 4 patients with PVR higher than 100 mL (22.2% of patients with voiding dysfunction), all with a maximal flow rate less than or equal to 12 mL/s. Table-3 displays statistically significant associations between the presence of voiding dysfunction and VS-Open and VS-Directed. Due to small numbers we are unable to describe variation in voiding symptoms according to the time elapsed since the surgery. There was a statistically significant association between voiding dysfunction and a) the presence of any VS-Open and b) straining to void obtained by open anamnesis. There was no statistically significant association between voiding dysfunction and VS-Directed. Table-4 shows sensitivity, specificity, PPV, NPV, accuracy, positive and negative likelihood ratio and strength of agreement (Cohen’s kappa) of symptoms with statistically significant association.

**Table 1:** Medical history in women with previous anti-incontinence surgery undergoing urodynamics (n = 91).

Variable	Results
Age (range)	62.7 ± 11.06 (34 – 81)
Vaginal deliveries (range)	2.79 ± 1.91 (0 – 11)
Previous hysterectomy	31 (35%)
**Type of anti-incontinence surgery**	
Mid-urethral sling	50 (54.9%)
Burch colposuspension	29 (31.9%)
Mid-urethral sling and Burch surgery	5 (5.5%)
Unknown vaginal surgeries	7 (7.7%)
**Symptoms of**	
Stress urinary incontinence	11 (12.1%)
Urge urinary incontinence	20 (22.0%)
Mixed urinary incontinence	54 (59.3%)
Other types of urinary incontinence ^a^	6 (6.6%)
**VS-Open** ^**b**^	23 (25.3%)
Slow stream	5
Straining to void	9
Intermittent stream	13
Feeling of incomplete emptying ^c^	3
**VS-Directed** ^**d**^	70 (76.9%)
Slow stream	20
Straining to void	15
Intermittent stream	24
Feeling of incomplete emptying ^c^	45
Without any voiding symptom	21 (23.1%)

**a** = Insensible urinary incontinence, nocturnal enuresis; **b** = VS-Open: voiding symptoms obtained by open anamnesis; **c** = A post micturition symptom according to ICS; **d** = VS-Directed: voiding symptoms obtained by directed anamnesis

**Table 2:** Urodynamics results in women with previous anti-incontinence surgery according to the type of urinary incontinence symptoms (n = 91).

Type of symptom	Filling cystometry		Pressure-flow study	
Stress urinary incontinence (n = 11)	Urodynamic stress incontinence	11 (100%)	Bladder outlet obstruction	2(18%)
Urge urinary incontinence ^a^ (n = 20)	Detrusor overactivity	11 (55%)	Bladder outlet obstruction Mixed voiding dysfunction	4(20%) 1(5%)
Mixed urinary incontinence (n = 54)	Urodynamic stress incontinence Detrusor overactivity Mixed filling diagnosis ^b^	21(39%) 4(7.4%) 27(50%)	Bladder outlet obstruction Reduced detrusor contractility Mixed voiding dysfunction	4(7.4%) 3(5.6%) 1(1.9%)
Other types of urinary incontinence (n = 6)	Urodynamic stress incontinence	3 (50%)	Bladder outlet obstruction	3(50%)

**a** = In patients only with urge urinary incontinence symptoms the stress test wasn’t done; **b** = Mixed filling diagnosis: Urodynamic stress incontinence + Detrusor overactivity

**Table 3:** Statistical associations between voiding dysfunction and voiding symptoms in women with previous anti-incontinence surgery.

Symptom	p value
VS-Open ^a^	
Any spontaneous symptom	0.037
Slow stream	0.256
Straining to void	0.013
Intermittent stream	0.068
Feeling of incomplete emptying ^b^	0.488
VS-Directed ^c^	
Any directed symptom	0.551
Slow stream	0.072
Straining to void	0.179
Intermittent stream	0.368
Feeling of incomplete emptying ^b^	0.317

**a** = VS-Open: voiding symptoms obtained by open anamnesis; **b** = A post micturition symptom according to ICS; **c** = VS-Directed: voiding symptoms obtained by directed anamnesis

**Table 4:** Voiding symptoms as predictors of voiding dysfunction in women with previous anti-incontinence surgery.

	Any voiding symptom by open anamnesis	Straining to void by open anamnesis
Sensitivity	44.4%	27.8%
Specificity	80.8%	94.5%
PPV	36.3%	55.6%
NPV	85.5%	84.1%
Accuracy	73.6%	81.3%
Positive likelihood ratio	2.324	5.129
Negative likelihood ratio	0.686	0.764
Kappa (95% CI)	0.233 (0.006–0.460)	0.275 (0.029–0.521)

**PPV** = Positive predictive value; **NPV** = Negative predictive value; **CI** = Confidence interval

## DISCUSSION

This exploratory study, despite being retrospective and that included a limited number of patients, has the strength to evaluate a homogeneous group of women in a standardized manner, with documentation of analyzed data upon examination, following the definitions and recommendations of the IUGA and the ICS.

Correlation between voiding and/or post-micturition symptoms and voiding dysfunction is difficult to assess due to a lack of consensus in the definition and diagnosis of voiding dysfunction and in the way symptoms are retrieved.

For the diagnosis of voiding dysfunction, some studies use only the criterion of decreased urinary flow rate (5) or just the increase in PVR criterion (6-8). Other studies use both criteria but independently of one another (11, 19), and this modifies all the results obtained. Additionally, there is another group of studies that only considers the bladder outlet obstruction diagnosis, without assessing those patients with a reduced detrusor contractility, thus having an impact on conclusions (10, 12, 20). The present study follows the IUGA and ICS definition, and therefore the voiding dysfunction diagnosis considers both a decreased urinary flow criterion as well as the criterion of increased PVR. However, any definition of voiding dysfunction in females has a certain degree of arbitrariness. We chose the criteria of maximal flow rate ≤12 mL/s based on the main studies that use such cutoff to define bladder outlet obstruction in women when associated to high detrusor pressure at maximum flow rate (17, 21), that additionally coincides with the cutoff to define a reduced detrusor contractility when associated to low detrusor pressure at maximum flow rate described by Gotoh et al. (18). With regard to PVR, the IUGA/ICS joint report on the terminology for female pelvic floor dysfunction indicates different values for the upper limit of normal (30, 50 and 100 mL) (13). In the present study a value higher than 100 mL was chosen as abnormal. Such value is observed in only 5% of asymptomatic peri and post-menopausal women (22). Anyhow, in the present study, all patients with increased PVR had decreased maximal flow rates, therefore the results would not have been affected if higher PVR criterion had been defined, a fact that cannot be guaranteed in studies that do not consider both criteria.

To be able to compare our results, focus must be only on the scant studies that diagnose voiding dysfunction with urinary flow rate and PVR criteria, and that also include not only bladder outlet obstruction but also reduced detrusor contractility. It is with this perspective that Groutz et al., applying a medical interview apparently in a directed manner to 206 women, assessed the presence of at least one voiding symptom (hesitancy, straining to void, weak or prolonged stream, intermittent stream, double voiding, feeling of incomplete emptying, reduction and positional changes to start or complete voiding) and concluded that voiding dysfunction defined as a maximal flow rate less than 12 mL/s and/or a PVR higher than 150 mL (present in 40 patients) could be found in women with and without suggestive symptoms (in 21.2% and 16.5% respectively) (9). On the other hand, Hubeaux et al., using 5 items of the Bristol Female Lower Urinary Tract Symptoms Questionnaire (hesitancy, straining to void, intermittency, strength of urine stream, feeling of incomplete bladder emptying) in 93 women with genuine stress urine incontinence with no evident obstruction cause undergoing urodynamic testing, did not find an association with voiding dysfunction defined as a maximal flow rate less than 15 mL/s and/or a PVR higher than 50 mL and an abnormal pattern of the flow curve (23). Our study, that considered voiding dysfunction as the presence of a maximal flow rate less than or equal to 12 mL/s and/or a PVR higher than 100 mL, failed to find an association with VS-Directed, concurring with the study of Groutz et al., and similarly to the results of Hybeaux et al., if we consider that there is a similarity between asking about the presence of each symptom in a directed manner and applying a questionnaire that includes them all. Noteworthy, a reasonable association was found between voiding dysfunction and the presence of “any VS-Open” and with “straining to void obtained by open anamnesis” (Table-4). It is interesting to comment that Jeffery et al. also described the importance of the symptom “straining to void” as a predictor of voiding dysfunction, although they evaluated separately the maximal flow rate and the PVR. Through a standardized questionnaire applied to 116 patients, they evaluated the presence of voiding symptoms that occurred “more commonly than occasionally” (straining to void, double voiding, post-micturition leakage, slow urine stream and feeling of incomplete bladder emptying), and found that “straining to void” was the only predictor of decreased maximal flow rate (less than 15 mL/s) and of increased PVR (PVR higher than 100 mL and 150 mL) (11).

If the outcomes would be applied in the clinical practice, the “straining to void obtained by open anamnesis” almost ensures the diagnosis of voiding dysfunction (94.5% specificity, which is independent of the voiding dysfunction prevalence). If we generalize our results, in patients with overactive bladder syndrome, this symptom would be useful to decide a full urodynamic study to clarify the reason of the voiding dysfunction, considering that a bladder outlet obstruction can cause detrusor overactivity (4) and that antimuscarinc therapy in patients with voiding dysfunction may deteriorate their clinical conditions. As well as, patients with stress-predominant urinary incontinence, this symptom would help to determinate the need of a full urodynamic study to evaluate voiding dysfunction, which is associated with obvious worse surgical outcomes (the ValUE trial reported that 11.9% of the patients of the urodynamic-testing group had voiding dysfunction despite having a PVR less than 150 mL and that these patients had less satisfactory outcomes (62.1% vs. 78.3%) (24). Finally, we have to be careful with the interpretation of the high NPV of “any VS-Open” and “straining to void obtained by open anamnesis”: without considering any voiding symptom our cohort has a 80.2% probability of not having voiding dysfunction (19.8% patients with voiding dysfunction), which increases only to 85.5% if the patient reports “any VS-Open” and to 84.1% if the patient reports “straining to void by open anamnesis”.

## CONCLUSIONS

This study shows that VS-Open may predict better voiding dysfunction than VS-Directed in women. To date, we are not aware of prior publications having studied this matter. Additional larger and prospective studies are required to confirm these findings.
